# Spectrum of Movement Disorders in Niemann-Pick Disease Type C

**DOI:** 10.5334/tohm.701

**Published:** 2022-09-08

**Authors:** Rashmi Devaraj, Rohan R. Mahale, D. M. Sindhu, Albert Stezin, Nitish Kamble, Vikram V. Holla, M. Netravathi, Ravi Yadav, Pramod Kumar Pal

**Affiliations:** 1 Department of Neurology, National Institute of Mental health and Neurosciences, Bengaluru, India-560029

**Keywords:** Niemann-Pick Type C, movement disorders, cerebellar ataxia, dystonia, supranuclear gaze palsy, sphingolipidoses

## Abstract

**Introduction:**

Niemann-Pick disease type C (NPC) is an autosomal recessive neurovisceral lipid storage disorder caused by mutations in the *NPC 1* or *2* genes. Movement disorders can occur as the first symptom and as predominant symptom mainly in juvenile-onset. The frequency and heterogeneity of movement disorders in NPC are not well described. We studied the frequency and spectrum of movement disorders in patients with NPC of different age of onset.

**Methods:**

Retrospective chart review of patients with NPC diagnosed based on the Suspicion Index tool and demonstration of foamy macrophages/sea-blue histiocytes in bone marrow aspirate.

**Results:**

We report 9 cases of NPC with 2 patients of late-infantile, 4 juvenile-onset and 3 of adult-onset. The mean age at onset of symptoms was 11.7 ± 10.4 (range 4–38 years) and the median duration of illness was 4 years. Vertical supranuclear gaze palsy (VSGP) was noted in 8 patients and VSGP with slowing of saccade in 1 patient. Splenomegaly was seen in 5 patients. Movement disorders as the first symptom occurred in 4 patients. Dystonia was the first symptom in 2 patients and cerebellar ataxia in 2 patients. Cerebellar ataxia occurred during the course of illness in 5 patients, dystonia in 6 patients. One patient with late-infantile NPC had stimulus-sensitive myoclonus.

**Conclusion:**

Movement disorders are common in NPC and occur as a presenting symptom. Cerebellar ataxia and dystonia are the most common movement disorder in NPC. Vertical supranuclear gaze palsy along with the movement disorders should lead to clinical suspicion of NPC.

## 1. Introduction

Sphingolipidoses are the lysosomal storage diseases that occur due to the defective degradation and accumulation of sphingolipids [1,2,3]. Niemann-Pick (NP) diseases are the sphingolipidoses named after the German physicians Albert Niemann and Ludwick Pick who first described the disorders. There is accumulation of sphingomyelin due to the defect in the enzyme sphingomyelinase. NP Type A (NPA) and NP Type B (NPB) are caused by defects in sphingomyelinase, whereas sphingomyelinase activity is normal in NP Type C (NPC) [4,5,6]. NPC is characterised by the intracellular accumulation of low-density lipoprotein-derived, unesterified cholesterol and sphingosine. It is due to the mutations in the NPC1 (95% of cases) or NPC2 (5%) genes with autosomal recessive inheritance [7,8]. NPC1 and NPC2 are the proteins involved in the transport of unesterified cholesterol and sphingosine out of the late endosome/lysosome compartment [9,10]. NPC has a wide clinical spectrum that ranges from a fatal antenatal disorder to an adult-onset chronic neurodegenerative disease. Based on the age of onset of neurological manifestations, it is classified as the early-infantile (<2 years), late-infantile (2–6 years), juvenile (6–15 years) and adult (>15 years) onset NPC [11,12]. The late infantile and the juvenile forms of NPC constitutes about 60–70% of all cases of NPC. The diagnosis of NPC is based on the combination of biochemical and molecular genetic studies. The plasma biomarkers currently in use are oxysterols, lyso-SM-509 and lyso-sphingomyelin [13]. The confirmation of the diagnosis of NPC is by mutation analysis of *NPC1* and *NPC2* genes [14]. Filipin test is no longer recommended as a first line test for the diagnosis of NPC [15]. But it is a useful diagnostic tool in uncertain cases wherein the biomarkers and/or molecular analysis is inconclusive [14]. The commonest neurological manifestation in NPC is cerebellar ataxia, vertical supranuclear gaze palsy (VSGP), dysarthria, dysphagia, dementia and movement disorders [12]. Cerebellar ataxia, dystonia and myoclonus are the common movement disorder reported in NPC [16]. The knowledge about the frequency, heterogeneity and timing of movement disorders during the course of the illness is sparse. We aimed to characterize the spectrum of movement disorders and additional other systemic involvement, blood investigations, and imaging abnormalities in NPC. This study will add to the growing knowledge about the frequency and spectrum of movement disorders in NPC.

## 2. Subjects and Methods

### Subject recruitment and clinical evaluation

This was a retrospective chart review of patients with NPC who were evaluated in the neurology out-patient department, and movement disorder clinic from January 2010 to December 2020 at the National Institute of Mental Health and Neurosciences, Bengaluru. The diagnosis of NPC was made based on the Suspicion Index tool [17] and demonstration of foamy macrophages/sea-blue histiocytes in bone marrow aspirate or presence of mutation in the *NPC1* or *NPC2* genes. The Filipin test [15], plasma biomarkers [13] and molecular genetic testing (in majority of cases) were not available. The Suspicion index tool consists of total risk prediction score. The total risk prediction score of <40 have a low probability of having NPC; a score of 40–69 indicates that further follow-up observation is required, and score of >70 have high probability. Patients with Suspicion index tool total risk prediction score of >70 and presence of foamy macrophages/sea-blue histiocytes in bone marrow aspirate or positive mutation in *NPC1* or *NPC2* genes were included for the analysis. Institute Ethics Committee approval (No:NIMH/DO/DEAN (Basic Science)/2020–21) was obtained for the retrospective analysis of the data and the informed consent was obtained from the patients for video recording and publishing. Patients’ details were anonymized to maintain patient privacy. A detailed review of all the charts was done. The demographic details, age at onset, duration of the illness, clinical features, family history of similar illness, consanguinity, clinical examination findings and investigations including blood investigations, imaging and genetic data (where available) were collected. The videos of the patients were reviewed for detailed description of the movement disorder.

### Statistical analysis

Data was expressed using descriptive statistics. Continuous variables were expressed as mean/median with standard deviation/Inter-quartile range respectively whereas categorical variables were expressed as frequencies and percentages.

## 3. Results

### Demographic data

Nine patients (6 males; 3 females) with suspicion index tool score >70 were included in the study. The mean age at presentation was 16.8 ± 12.1 years (median: 20 years, range 6–45 years) and mean age at onset was 11.7 ± 10.4 years (median: 13 years, range 4–38 years). The mean duration of the illness, from the time of onset of symptoms to the evaluation at our hospital was 4.1 ± 2.7 years (median: 4 years, range- 2–17 years). One patient had consanguineous parentage and one patient had similar clinical features in 2 siblings with age at onset at 30–40 years. Based on the age of onset, 2 patients had late-infantile onset, 4 had juvenile-onset and 3 had adult-onset NPC.

### Clinical features

Movement disorders as the first symptom occurred in 4 patients. Dystonia was the first symptom in 2 patients (one with late-infantile and one with juvenile-onset) and cerebellar ataxia in 2 patients (one with juvenile-onset and one with adult-onset). The initial symptoms in other patients were behavioural changes in the form of disinhibition, anger outburst in 2 patients with adult-onset type, regression of milestones with stimulus-sensitive myoclonus in 1 patient with late-infantile onset and decline in cognition in 2 patients with juvenile-onset NPC. Cerebellar ataxia occurred during the course of illness in additional 3 patients (3 patients with adult-onset type and 2 patients with juvenile-onset type had cerebellar ataxia in total). Dystonia occurred during the course of illness in total 6 patients and it was generalised dystonia in 2 patients, bilateral finger dystonia in 3 patients and cervical dystonia in 1 patient. VSGP was seen in 8 patients and one patient with adult-onset had VSGP with only slowing of vertical saccades. Head thrust was seen in 2 patients (1 with late- infantile and 1 with juvenile-onset). Splenomegaly was seen in 5 patients. Two patients had splenomegaly detected both clinically and on ultrasound. Remaining 3 patients had splenomegaly detected on ultrasound only. There was no hepatomegaly on ultrasound in any of the 9 patients. One patient with juvenile-onset NPC had history of neonatal jaundice. The clinical and radiological details of each case is shown in Table 1.

**Table 1:** Clinical and radiological details of cases with preference to movement disorders.


CASE	AGE AT ONSET (YEARS)	AGE AT DIAGNOSIS (YEARS)	FIRST SYMPTOM	CEREBELLAR ATAXIA	DYSTONIA	MYOCLONUS	COGNITIVE DISTURBANCE	BEHAVIORAL CHANGE	BRAIN IMAGING FINDINGS

1	4	6	Regression of milestones	–	–	+	+	–	Fronto-temporal cerebral atrophy with PV- WMH

2	25	27	Cerebellar ataxia	+	+ (finger dystonia)	–	–	–	Normal

3	38	45	Behavioral change	+	+ (finger dystonia)	–	+	+	Diffuse cerebral and cerebellar atrophy

4	20	24	Behavioral change	+	+ (finger dystonia)	–	+	+	Diffuse cerebral and cerebellar atrophy

5	12	20	Dystonia	–	+ (Generalised)	–	+	–	Diffuse cerebral and cerebellar atrophy

6	4	8	Dystonia	–	+ (Generalised)	–	+	–	Not available

7	7	9	Ataxia with slow eye movements	+	+ (cervical)	–	+	–	Mild cerebral and cerebellar atrophy

8	13	20	Reduced cognition	+	–	–	+	–	Diffuse cerebral and cerebellar atrophy

9	15	32	Reduced cognition	–	–	–	+	–	Diffuse cerebral and cerebellar atrophy


+/– present/absent; PV-WMH- periventricular white mater hyperintensities.

### Neuroimaging

**Figure 1:**
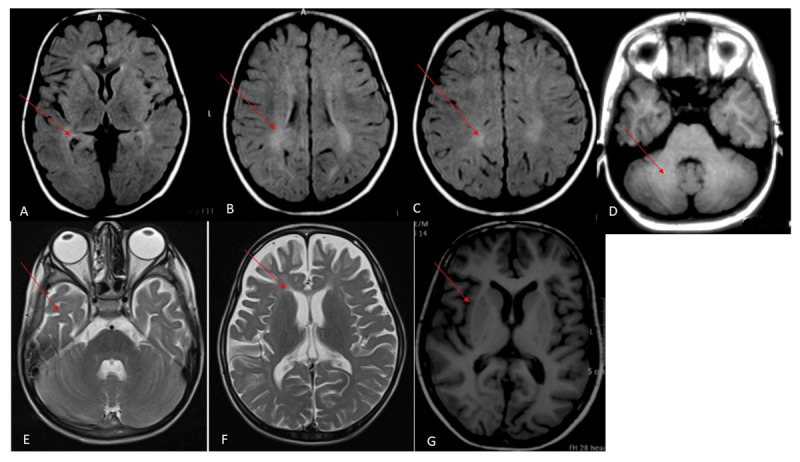
Brain MRI of 2 patients with late-infantile onset NPC (A-F); **(A–C)** Fluid-attenuated inversion recovery (FLAIR) axial images showing periventricular hyperintense signals (red arrow); **(D)** axial T1-weighted image showing normal cerebellum (red arrow); **(E, F)** Axial T2-weighted image showing periventricular hyperintense signals and fronto-temporal atrophy (red arrow); **(G)** Axial T1-weighted image of juvenile-onset NPC showing right lateral frontal atrophy (red arrow).

**Figure 2:**
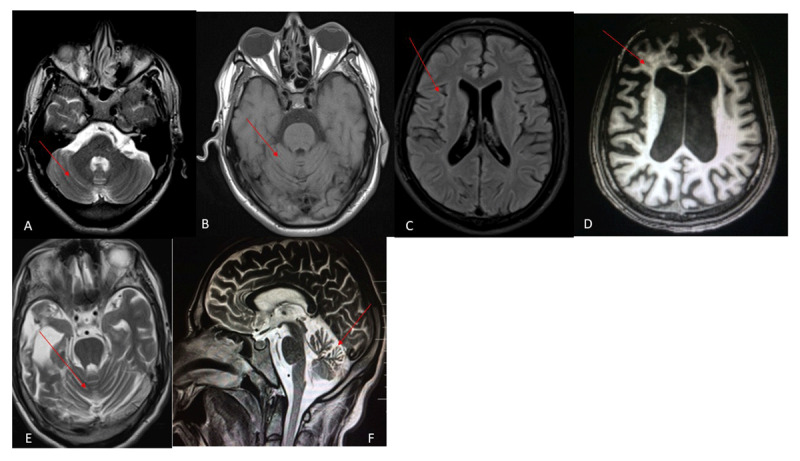
Brain MRI of patients with adult-onset NPC; Patient no 2 **(A)** Sagittal T2-weighted image showing cerebellar atrophy (red arrow); Patient no 3 (B, C)- **(B)** Axial T1-weighted image showing frontal atrophy with prominent ex-vacuo dilation of lateral ventricles (red arrow); **(C)** Axial T2-weighted image showing bilateral temporal atrophy and cerebellar atrophy (red arrow); Patient no 4 (D-F); **(D)** Axial T2-weighted image showing cerebellar atrophy (red arrow); **(E)** Axial T1-weighted image showing cerebellar atrophy (red arrow); **(F)** Axial FLAIR image showing cortical atrophy (red arrow).

Brain magnetic resonance imaging (MRI) was done in 8 patients. Diffuse cerebral and cerebellar atrophy was seen in 6 patients. Two patients with late-infantile onset had signal change in periventricular white matter with bilateral fronto-temporal atrophy (Figures 1 & 2). Computed tomography of the head was done in one patient with juvenile-onset and showed cerebellar atrophy.

### Bone marrow biopsy and haematological findings

**Figure 3:**
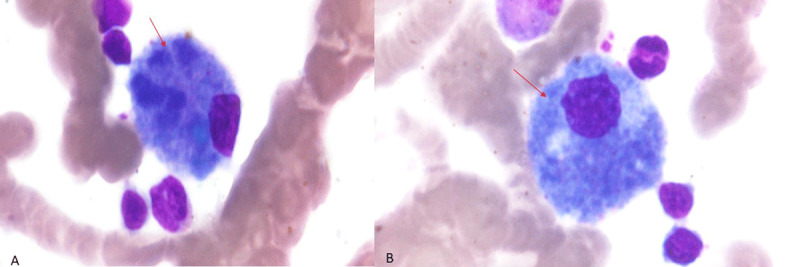
Bone marrow aspirate **(A, B)** showing sea blue histiocytes (red arrow).

Bone marrow biopsy was done in 6 patients and showed foamy macrophages and sea blue histiocytes (Figure 3). The other 3 patients without bone marrow biopsy had genetic confirmation of NPC. There was thrombocytopenia in 5 patients (3 patients with adult-onset, 1 patient with late-infantile onset and 1 patient with juvenile-onset).

### Genetic analysis

Genetic analysis was done in 3 patients with juvenile-onset NPC. A novel homozygous missense variation in exon 16 of the *NPC1* gene (NM_000271:c.2473T>C: p.Tyr825His) in patient 5. Two likely pathogenic missense variants in compound heterozygous state in *NPC1* gene (ENST00000269228:c.2974G>T;p.Gly992Trp/c.3634G>T’p.Val1212Leu) occurred in patient 8. A likely pathogenic missense variant in homozygous state in NPC2 gene (ENST00000541064:c.358C>A:p.Pro120Thr) was seen in patient 9.

### Case vignettes

#### Patient-2 (Adult NPC)

A 27-year-old lady developed imbalance while walking of 2 years duration which was slowly progressive. At presentation, she was self-ambulatory. She had vertical saccades slowing, dystonic posturing of fingers (Video 1), impaired tandem gait. There was mild splenomegaly with thrombocytopenia. MRI of the brain was normal. Bone marrow examination showed sea blue histiocytes and foamy macrophages.

**Video 1:** **Adult-onset NPC.** Video of patient-2 showing slow vertical saccades with round the houses sign, zig-zag trajectory of vertical saccades (segment 1) and outstretched hands finger dystonia (segment 2) (Consent taken for publication).

#### Patient-5 (Juvenile NPC)

A 20-year-old lady born of non-consanguineous parental. She had a history of neonatal jaundice and developed dystonia of upper limbs at the age of 12 years which became generalised involving cranial, oropharyngeal musculature. The dystonia was gradual in onset and progressed to be generalised in topographic distribution within 3 years of onset. In addition, there was VSGP. The Burke-Fahn-Marsden dystonia rating scale (BFMDRS) score was 109. MRI of the brain showed diffuse cerebral and cerebellar atrophy. She underwent deep brain stimulation (DBS) of bilateral globus pallidus interna (GPi) due to sub-optimal response to medications. At 6-month follow-up, there was reduction in the severity of dystonia (BFMDRS score – 68). She was able to ambulate with persons support (Video 2).

**Video 2:** **Juvenile-onset NPC.** Video of patient-5 showing generalized dystonia (segment 1) and vertical supranuclear gaze palsy (segment 2). There was no parkinsonism (Consent taken for publication).

#### Patient-6 (late infantile NPC)

An 8-year-old girl child born out of consanguineous parentage with normal perinatal history and developmental milestones developed dystonia of upper limbs at the age of 4 years which became generalised within 1–2 years. There was no diurnal variation. She had decrease in scholastic performance with dysarthria. She had VSGP, generalised dystonia and bi-pyramidal signs (Video 3). Bone marrow examination showed sea blue histiocytes.

**Video 3:** **Late infantile onset NPC.** Video of patient-6 showing vertical saccadic initiation failure with head thrust (segment 1) and upper and lower limb dystonia (segment 2) (Consent taken for publication).

## 4. Discussion

Movement disorders is one of the clinical manifestations of NPC. We report 9 cases of NPC with different age at onset and elucidate the spectrum of movement disorders in NPC. Two patients had late-infantile onset, 4 juvenile-onset and 3 adult-onset NPC. Movement disorders was the first symptom in 4 patients. Dystonia was the most common movement disorder in our cohort followed by ataxia and myoclonus. The spectrum of movement disorders in late-infantile NPC has not been reported. There are few studies which have described the spectrum of movement disorders in juvenile and adult-onset NPC. Ebrahimi-Fakhari et al reported spectrum of movement disorders in 5 patients with NPC. The age of onset of movement disorder was between 10 and 14 years of age. Ataxia and generalised dystonia (4 out of 5 patients) and only ataxia in one patient was the most common movement disorder in their cohort. Gelastic cataplexy was reported in 2 patients and akathisia in 1 patient [18]. Anheim et al from France reported the heterogeneity of movement disorders in 4 juvenile-onset and 1 adult-onset NPC patients. Ataxia was the most common movement disorder followed by dystonia, myoclonus and chorea [19]. Koens et al reported 8 cases of NPC wherein 5 were juvenile-onset and 3 adolescent/adult-onset NPC. Myoclonus (cortical, positive and negative) and dystonia (generalised) were the most common movement disorder followed by ataxia [20]. Overall, dystonia, ataxia and myoclonus are the most common movement disorder in NPC across all age of onset. A summary of these studies is shown in Table 2.

**Table 2:** Summary of other studies on spectrum of movement disorders in NPC.


STUDY	SEVIN ET AL, 2006	ANHEIM ET AL, 2014	KOENS ET AL, 2016	CURRENT STUDY

Number of patients	13	5	8	9

Study country	France	France	Netherlands	India

Categorization based on age at onset	13 adult-onset	4 juvenile-onset 1 adult-onset	5 juvenile-onset 3 adolescent/adult-onset	2 late-infantile onset 4 juvenile-onset 3 adult-onset

Types of movement disorders (n)	Ataxia (11) Dystonia (7) Myoclonus (2) Chorea (1) Cataplexy (2)	Ataxia (5) Dystonia (3) Myoclonus (3) Chorea (1)	Myoclonus (5) Dystonia (5) Ataxia (3)	Dystonia (6) Ataxia (5) Myoclonus (1)

Movement disorder as first symptom (n)	Ataxia (2) Dystonia (3)	Ataxia (3) Myoclonus (1)	Ataxia (4) Myoclonus (3) Dystonia (1)	Ataxia (2) Dystonia (2)


n-number of patients, NPC-Niemann-Pick Type C.

The movement disorders in adult-onset NPC in our cohort were cerebellar ataxia in all 3 patients (as presenting symptom in 1 patient) and bilateral finger dystonia in 3 patients. Sévin et al reported a larger cohort of 13 cases of adult-onset NPC and cerebellar ataxia, dystonia was the common movement disorder. Dystonia was generalised in 2 patients, hemidystonia in 1 patient and involving both hands and feet in 6 patients. Five patients had movement disorder as the first symptom (Table 2) [16]. Anheim et al reported 1 patient with adult-onset NPC presenting with cerebellar ataxia and cortical myoclonus [19]. Koens et al reported 3 patients with adult-onset and 2 had ataxia, 1 had myoclonus as the first symptom. All 3 patients had cerebellar ataxia, 2 patients had generalised dystonia and 2 patients had myoclonus during the course of their illness [20].

Juvenile-onset patients of our cohort had generalised dystonia as the first symptom in 1 patient and cerebellar ataxia in 1 patient. Ebrahimi-Fakhari et al reported 3 patients with juvenile-onset and all 3 had cerebellar ataxia and generalised dystonia [18]. Anheim et al reported 4 patients with juvenile-onset and cerebellar ataxia occurred in all 4 patients, dystonia in 3, myoclonus in 2 and chorea in 1 patient. Cerebellar ataxia was the first symptom in 3 patients and myoclonus in 1 patient in their cohort [19]. Koens et al reported 5 patients with juvenile-onset and myoclonus in 2 patients, cerebellar ataxia in 2 patients and dystonia in 1 patient was the presenting movement disorder. Cerebellar ataxia was seen overall in 4 patients, dystonia in 3 and myoclonus in 3 patients [20]. One patient with juvenile-onset NPC from our cohort had undergone bilateral GPi DBS for the generalised dystonia with improvement in the severity of the dystonia. Gonzalez V et al had reported DBS (GPi and thalamic target) in one patient with adolescent-onset NPC who had status dystonicus [21]. DBS has shown to reduce the severity of dystonia in NPC.

Jahnova et al reported 13 patients with late-infantile onset NPC and had psychomotor retardation/regression, retardation of speech development, epilepsy, gelastic seizures/cataplexy, ataxia and dysarthria [22]. We had 2 patients with late-infantile onset and one patient presented with generalised dystonia and the other with regression of acquired milestones with stimulus-sensitive myoclonus. To summarise the spectrum of movement disorders in different age of onset, juvenile and adult-onset NPC have similar spectrum of dystonia, ataxia and myoclonus whereas late-infantile onset NPC have dystonia and myoclonus associated with developmental delay than the cerebellar ataxia.

Vertical supranuclear saccadic paresis is one of the robust early neurological indicators of NPC. Slow and hypometric vertical saccades particularly downward is the initial oculomotor abnormality in NPC. As the disease progresses, there is complete VSGP seen [23,24]. All our patients had VSGP except one patient who had VSGP with only slowing of saccades. Interestingly, we had 4 patients with thrombocytopenia which may be secondary to splenic sequestration. The thrombocytopenia has been reported in NPB cases and fetal NPC but not in the other age at onset. There are about 700 *NPC1* variants which have been reported so far, among which around 420 are considered as pathogenic variants. The p.I1061T *NPC1* mutation has been commonly associated with a juvenile-onset NPC and p.P1007A *NPC1* mutation commonly associated with a juvenile and adult onset [25].

This study adds to the growing literature on the spectrum of movement disorders in different age at onset cases of NPC. However, this study was not devoid of limitations. The small sample size, lack of molecular genetic testing for diagnostic confirmation, selection and recall bias while recruiting the cases were the limitations of this study.

## Conclusion

Movement disorders are common in NPC and it can occur as a presenting symptom. Dystonia and cerebellar ataxia were the most common movement disorder presenting as first symptom in our cohort. However, dystonia was the most common movement disorder during the course of the illness. NPC has variable clinical presentation according to the age of onset. Juvenile and adult-onset NPC have similar spectrum of movement diorders with dystonia, ataxia and myoclonus as the common movement disorder. However, late-infantile have dystonia and myoclonus as common movement disorder than cerebellar ataxia associated with developmental delay. The presence of VSGP, movement disorders, dysarthria, dysphagia, cognitive decline and psychiatric disturbances should lead to the clinical suspicion of NPC.

## References

[1] Vitner EB, Platt FM, Futerman AH. Common and uncommon pathogenic cascades in lysosomal storage diseases. J Biol Chem. 2010; 285: 20423–7. DOI: 10.1074/jbc.R110.134452 20430897 PMC2898325

[2] Coant N, Sakamoto W, Mao C, Hannun YA. Ceramidases, roles in sphingolipid metabolism and in health and disease. Adv Biol Regul. 2017; 63: 122–31. DOI: 10.1016/j.jbior.2016.10.002 27771292 PMC5330250

[3] Grassi S, Chiricozzi E, Mauri L, Sonnino S, Prinetti A. Sphingolipids and neuronal degeneration in lysosomal storage disorders. J Neurochem. 2019; 148: 600–11. DOI: 10.1111/jnc.14540 29959861

[4] Brady RO, Kanfer JN, Mock MB, Fredrickson DS. The metabolism of sphingomyelin. II. Evidence of an enzymatic deficiency in Niemann-Pick diseae. Proc Natl Acad Sci U S A. 1966; 55: 366–9. DOI: 10.1073/pnas.55.2.366 5220952 PMC224150

[5] Vanier MT, Revol A, Fichet M. Sphingomyelinase activities of various human tissues in control subjects and in Niemann-Pick disease – development and evaluation of a microprocedure. Clin Chim Acta. 1980; 106: 257–67. DOI: 10.1016/0009-8981(80)90309-5 6251986

[6] Neufeld EB, Wastney M, Patel S, Suresh S, Cooney AM, Dwyer NK, et al. The Niemann-Pick C1 protein resides in a vesicular compartment linked to retrograde transport of multiple lysosomal cargo. J Biol Chem. 1999; 274: 9627–35. DOI: 10.1074/jbc.274.14.9627 10092649

[7] Carstea ED, Morris JA, Coleman KG, Loftus SK, Zhang D, Cummings C, et al. Niemann-Pick C1 disease gene: homology to mediators of cholesterol homeostasis. Science. 1997; 277: 228–31. DOI: 10.1126/science.277.5323.228 9211849

[8] Naureckiene S, Sleat DE, Lackland H, Fensom A, Vanier MT, Wattiaux R, et al. Identification of HE1 as the second gene of Niemann-Pick C disease. Science. 2000; 290: 2298–301. DOI: 10.1126/science.290.5500.2298 11125141

[9] Benussi A, Cotelli MS, Padovani A, Borroni B. Recent neuroimaging, neurophysiological, and neuropathological advances for the understanding of NPC. F1000Res. 2018 Feb 15; 7: 194. DOI: 10.12688/f1000research.12361.1 29511534 PMC5814740

[10] Polese-Bonatto M, Bock H, Farias ACS, Mergener R, Matte MC, Gil MS, et al. Niemann-Pick Disease Type C: Mutation Spectrum and Novel Sequence Variations in the Human NPC1 Gene. Mol Neurobiol. 2019; 56: 6426–35. DOI: 10.1007/s12035-019-1528-z 30820861

[11] Patterson MC, Mengel E, Wijburg FA, Muller A, Schwierin B, Drevon H, et al. Disease and patient characteristics in NP-C patients: findings from an international disease registry. Orphanet J Rare Dis. 2013; 8: 12. DOI: 10.1186/1750-1172-8-12 23324478 PMC3558399

[12] Vanier MT. Niemann-Pick disease type C. Orphanet J Rare Dis. 2010 Jun 3; 5: 16. DOI: 10.1186/1750-1172-5-16 20525256 PMC2902432

[13] Jiang X, Sidhu R, Porter FD, et al. A sensitive and specific LC-MS/MS method for rapid diagnosis of Niemann-Pick C1 disease from human plasma. J Lipid Res. 2011; 52: 1435–45. DOI: 10.1194/jlr.D015735 21518695 PMC3122908

[14] Geberhiwot T, Moro A, Dardis A, Ramaswami U, Sirrs S, Marfa MP, et al; International Niemann-Pick Disease Registry (INPDR). Consensus clinical management guidelines for Niemann-Pick disease type C. Orphanet J Rare Dis. 2018; 13(1): 50. DOI: 10.1186/s13023-018-0785-7 29625568 PMC5889539

[15] Vanier MT, Latour P. Laboratory diagnosis of Niemann-Pick disease type C: the filipin staining test. Methods Cell Biol. 2015; 126: 357–75. DOI: 10.1016/bs.mcb.2014.10.028 25665455

[16] Sevin M, Lesca G, Baumann N, Millat G, Lyon-Caen O, Vanier MT, et al. The adult form of Niemann-Pick disease type C. Brain 2007; 130: 120–33. DOI: 10.1093/brain/awl260 17003072

[17] Wijburg FA, Sedel F, Pineda M, Hendriksz CJ, Fahey M, Walterfang M, et al. Development of Suspicion index to aid diagnosis of Niemann-Pick disease type C. Neurology 2012; 78: 1560–67. DOI: 10.1212/WNL.0b013e3182563b82 22517094

[18] Ebrahimi-Fakhari D, Hildebrandt C, Davis PE, Rodan LH, Anselm I, Bodamer O. The Spectrum of Movement Disorders in Childhood-Onset Lysosomal Storage Diseases. Mov Disord Clin Pract. 2018; 5: 149–155. DOI: 10.1002/mdc3.12573 29930972 PMC6005694

[19] Anheim M, Lagha-Boukbiza O, Fleury-Lesaunier MC, Valenti-Hirsch MP, Hirsch E, Gervais-Bernard H, et al. Heterogeneity and frequency of movement disorders in juvenile and adult-onset Niemann-Pick C disease. J Neurol. 2014; 261: 174–9. DOI: 10.1007/s00415-013-7159-9 24178705

[20] Koens LH, Kuiper A, Coenen MA, Elting JW, de Vries JJ, Engelen M, et al. Ataxia, dystonia and myoclonus in adult patients with Niemann-Pick type C. Orphanet J Rare Dis. 2016 Sep 1; 11(1): 121. DOI: 10.1186/s13023-016-0502-3 27581084 PMC5007743

[21] Gonzalez V, Cif L, Ayrignac X, Cyprien F, Nerrant E, Sanrey E, et al. Status dystonicus treated by Deep Brain Stimulation in Niemann Pick type C [abstract]. Mov Disord. 2017; 32 (suppl 2).

[22] Jahnova H, Dvorakova L, Vlaskova H, Hulkova H, Poupetova H, Hrebicek M, et al. Observational, retrospective study of a large cohort of patients with Niemann-Pick disease type C in the Czech Republic: a surprisingly stable diagnostic rate spanning almost 40 years. Orphanet J Rare Dis. 2014 Sep 19; 9: 140. DOI: 10.1186/s13023-014-0140-6 25236789 PMC4193985

[23] Salsano E, Umeh C, Rufa A, Pareyson D, Zee DS. Vertical supranuclear gaze palsy in Niemann-Pick type C disease. Neurol Sci. 2012; 33: 1225-32. DOI: 10.1007/s10072-012-1155-1 22810120

[24] Di Lazzaro V, Marano M, Florio L, De Santis S. Niemann-Pick type C: focus on the adolescent/adult onset form. Int J Neurosci. 2016; 126: 963–71. DOI: 10.3109/00207454.2016.1161623 26998855

[25] NP-C Guidelines Working Group, Wraith JE, Baumgartner MR, Bembi B, Covanis A, Levade T, Mengel E, et al. Recommendations on the diagnosis and management of Niemann-Pick disease type C. Mol Genet Metab. 2009; 98: 152–65. DOI: 10.1016/j.ymgme.2009.06.008 19647672

